# A novel balance training system using multimodal biofeedback

**DOI:** 10.1186/s12938-016-0160-7

**Published:** 2016-04-22

**Authors:** Muhammad Raheel Afzal, Min-Kyun Oh, Hye Young Choi, Jungwon Yoon

**Affiliations:** School of Mechanical and Aerospace Engineering & ReCAPT, Gyeongsang National University, Jinju, 52828 Republic of Korea; Department of Rehabilitation Medicine, Gyeongsang National University School of Medicine, Gyeongsang National University Changwon Hospital, Changwon, 51472 Republic of Korea; Department of Radiology, Gyeongsang National University School of Medicine, Gyeongsang National University Hospital, Jinju, 52727 Republic of Korea

**Keywords:** Multimodal biofeedback, Visual biofeedback, Haptic biofeedback, Postural stability, Balance training system

## Abstract

**Background:**

A biofeedback-based balance training system can be used to provide the compromised sensory information to subjects in order to retrain their sensorimotor function. In this study, the design and evaluation of the low-cost, intuitive biofeedback system developed at Gyeongsang National University is extended to provide multimodal biofeedback for balance training by utilization of visual and haptic modalities.

**Methods:**

The system consists of a smartphone attached to the waist of the subject to provide information about tilt of the torso, a personal computer running a purpose built software to process the smartphone data and provide visual biofeedback to the subject by means of a dedicated monitor and a dedicated Phantom Omni^®^ device for haptic biofeedback. For experimental verification of the system, eleven healthy young participants performed balance tasks assuming two distinct postures for 30 s each while acquiring torso tilt. The postures used were the one foot stance and the tandem Romberg stance. For both the postures, the subjects stood on a foam platform which provided a certain amount of ground instability.

**Results:**

Post-experiment data analysis was performed using MATLAB^®^ to analyze reduction in body sway. Analysis parameters based on the projection of trunk tilt information were calculated in order to ascertain the reduction in body sway and improvements in postural control. Two-way analysis of variance (ANOVA) showed no statistically significant interactions between postures and biofeedback. Post-hoc analysis revealed statistically significant reduction in body sway on provision of biofeedback. Subjects exhibited maximum body sway during no biofeedback trial, followed by either haptic or visual biofeedback and in most of the trials the multimodal biofeedback of visual and haptic together resulted in minimization of body sway, thus indicating that the multimodal biofeedback system worked well to provide significant (p < 0.05) assistance in postural control.

**Conclusions:**

A multimodal biofeedback system can offer more customized training methods and hence provide therapists with a comprehensive solution for a diverse array of patients. It is necessary to identify the long-term effects of this novel biofeedback system. In the future, the balance training schemes for individuals with upright balance issues will be studied.

## Background

Balance and postural control are of vital importance for a person to walk and perform daily activities in a safe manner. These capabilities can be weakened as a consequence of the aging process, disease or trauma. The depletion of these capabilities is dangerous and can lead to falls [1], resulting in injury. Musculoskeletal and neurological factors including the vestibular system, vision, proprioception, muscle strength, and cognition are involved, in a complex manner, in postural and balance control [2]. As a result of aging or trauma, the sensory information available for the performance of these tasks is reduced. In such cases, sensory augmentation through the use of biofeedback devices can be applied to compensate for this weakness. Various biofeedback systems have been developed to improve balance based on different modalities, such as visual biofeedback [3–6], auditory biofeedback [7–9], and haptic biofeedback [10–12].

In a healthy human being, visual cues are employed as anchors of the environment. Therefore in biofeedback systems visual modality is usually provided on a balance training system with a fixed environment, hence prohibiting the options of wear-ability and outdoor use. The auditory biofeedback and haptic (vibrotactile) biofeedback systems, however, have the advantage of being able to be employed as wearable balance assistance systems. In comparison to audio biofeedback, a kinesthetic haptic interaction based system can provide continuous assistance without the discomfort of getting noisy and becoming a source of irritation for the user. The term light touch refers to fingertip contact with another physical object. Light touch based haptic cues have been used to improve postural stability [13]. Sensorimotor information of body displacement provided through contact of the index finger with a stationary bar can be used to stabilize body and reduce its sway [13, 14]. Light touch as a therapeutic mechanism can be a useful option in balance rehabilitation [15]. Albertsen et al. [16] showed that a light handgrip on a stick aids postural stabilization; the light grip facilitates delivery of haptic cues under natural circumstances. Thus, recent studies have used light touch based haptic cues to improve postural stability and balance. When standing quietly, individuals can use visual information from a fixed visual environment to reduce postural sway. It is believed that by providing additional visual information, individuals can become more aware of their body’s displacements and orientation in space. Hence, visual biofeedback is found to be effective in reducing body sway among young healthy and older healthy subjects, stroke patients and multiple sclerosis patients [17–20]. Long-term training using multimodal head-mounted biofeedback system (visual, audio and vibrotactile) has been shown to improve balance metrics in healthy young and older subjects [21]. Effects of multimodal biofeedback (visual and a waist-mounted vibrotactile belt) have also been investigated for subjects with defects in the vestibular system [22]. In comparison with single biofeedback (visual), multimodal biofeedback (visual and tactile) has been recognized to provide better performance during multi-tasking [23]. Recently, the use of light touch as kinesthetic haptic biofeedback has been verified as an effective method to provide balance cues to young healthy adults and stroke victims [24]. Kinesthetic haptic interface may be utilized not only to deliver cues in order to reduce body sway during standing but also in balance training featuring virtual/augmented reality, affording the therapists more therapeutic options and improving development of cognition in the patients. Therefore, a prospect for the study of novel multimodal biofeedback that combines kinesthetic haptic and visual biofeedback is existent.

In this study, the design of a low cost kinesthetic haptic feedback based balance training system [24] is extended to make it a multimodal biofeedback balance trainer. The system is capable of assisting the user in reducing body sway by providing multimodal biofeedback (featuring both haptic and visual biofeedback) based on trunk tilt measured with the help of a smartphone attached to the subject’s body. The objective of this study is to observe the effects of this novel multimodal biofeedback system in reduction of body sway and to empirically test the hypothesis that multimodal biofeedback can provide better assistance in the performance of balancing tasks as compared to a single-mode biofeedback system.

## Methods

**Fig. 1:**
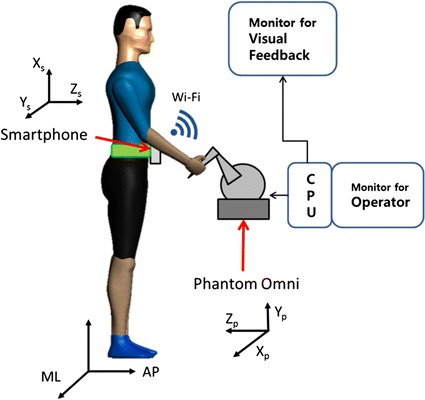
The conceptual diagram. The system features a waist-attached smartphone, software running on a computer (PC), a dedicated monitor for visual biofeedback and a dedicated Phantom Omni^®^ device for haptic biofeedback

The biofeedback system used in this study consists of four modules; a torso tilt measurement module, a data processing and control module, a visual biofeedback module and a kinesthetic biofeedback module. The torso tilt measurement module is composed of a smartphone which can be attached to the patient by means of an exclusive leather belt around the waist at L2-L4 lumbar spine region. Aforementioned torso tilt measurement module has been used as a reliable tool to assess body sway parameters during quite stance and gait motion [24, 25]. The smartphone (Pantech Vega IM-A850L [26]) continuously runs a dedicated Android application that measures the trunk tilt in terms of the mediolateral (ML) and anteroposterior (AP) angles, and sends the data to the data processing and control module through a “Socket” program communicating via Wi-Fi. The data processing and control module consists of a Personal Computer (PC) running a purpose-built program written in visual C++. Data sent by the smartphone is retrieved by the PC. The software running on the PC decodes the received data from the smartphone and generates corresponding outputs for providing visual and haptic biofeedback. The PC is connected with two display screens, one displaying information for the operator and the other functions as the visual biofeedback module (Fig. 1). The visual biofeedback display screen is placed in front of the test subject and displays a visual to help the subjects balance themselves. The kinesthetic biofeedback module consists of a haptic device which is connected to the PC. In our balance training system, the Phantom Omni^®^ is used [27], which is a commercially available low-cost haptic device. The smartphone used in this research featured a quad-core 1.5 GHz CPU with 2 GB of RAM, and efficiently ran the Android^®^ (Jelly Bean) operating system. The data bandwidth of the smartphone utilized here was 100 Hz. The system allowed for a torso tilt angle measurement resolution better than 0.1°.

The Phantom Omni^®^ can be connected to and controlled by a PC. The interface and implementation of Omni device for providing kinesthetic haptic biofeedback has been successfully tested [24]. The device can produce directional force in the X_*p*_, Y_*p*_ and Z_*p*_ directions; to ensure the virtual reference surface for the concept of light touch, the movement of the device handle in the Y_*p*_ axis was restrained, allowing motion of device handle in X_*p*_ Z_*p*_ plane only. An output force from the haptic device was always less than 1 N and the handle was allowed to deviate if any subject exerted a force larger than 1 N. Omni device’s handle maintained home position (0, 0, 0) at the beginning of haptic biofeedback. The ML and AP trunk tilt values were used to calculate the directions and magnitudes of all required forces; the handle then delivered these forces. The relationships between tilt angle and output haptic force magnitude and direction are given by Eqs. (1) and (2):

1 $$ F_{X} =  - k \times \left( {\frac{{trunk\;\;til{t_{ML}}}}{{range\;{X_P}}}} \right)\quad ({\rm{N}}) $$

2 $$F_{Z} = k \times \left({\frac{trunk\;\;til{t_{AP}}}{range\;{Z_{P}}}}
\right)\qquad ({\rm N})$$

where the “trunk tilt” is the tilt in ML or AP of the subject, calculated relative to the initial value as recorded at the start of the experiment, and the “range” in X_*p*_ and Z_*p*_ is the maximum permitted workspace (between −60 and +60 mm in both axes) of the haptic device [24]. The stiffness “k” was set to 0.05 N/mm to reduce jerkiness, thus providing smooth force transfer and not affecting the body sway. The trunk tilt information was also utilized to provide visual biofeedback on a LCD display screen attached to the PC. The screen was placed in front of the subject at head height at a distance of 1 m, in order to allow the subject to easily and comfortably maintain an upright posture while receiving focused feedback from the screen. Before the commencement of the experimental trials, the trunk angle feedback of the subject was represented as a circle at the center of the screen, the visual biofeedback during trials consisted of the motion of this circle in harmony with the trunk tilt variations captured from the waist mounted smartphone. The AP trunk tilt was mapped to the vertical motion of the circle and subsequently ML trunk tilt was mapped to the horizontal motion of the circle. The software generated this visual biofeedback at a refresh rate of 50 Hz (approximate latency of 40 ms) for display to the subjects. In order to provide multimodal biofeedback, both haptic biofeedback and visual biofeedback were provided simultaneously to the subjects.

Eleven healthy young subjects (9 males and 2 females, age 27.1 ± 3.1 years, weight 78.3 ± 6.6 kg, height 169.9 ±  9.2 cm) were recruited to check the effectiveness of biofeedback provided by our proposed system. None of the young healthy subjects had any history of sensorimotor or neurological disorders. These subjects did not suffer from any visual deficits other than adequately corrected loss of visual acuity. All of the subjects gave written informed consent in accordance with the rules of our local Ethics Committee.

**Fig. 2:**
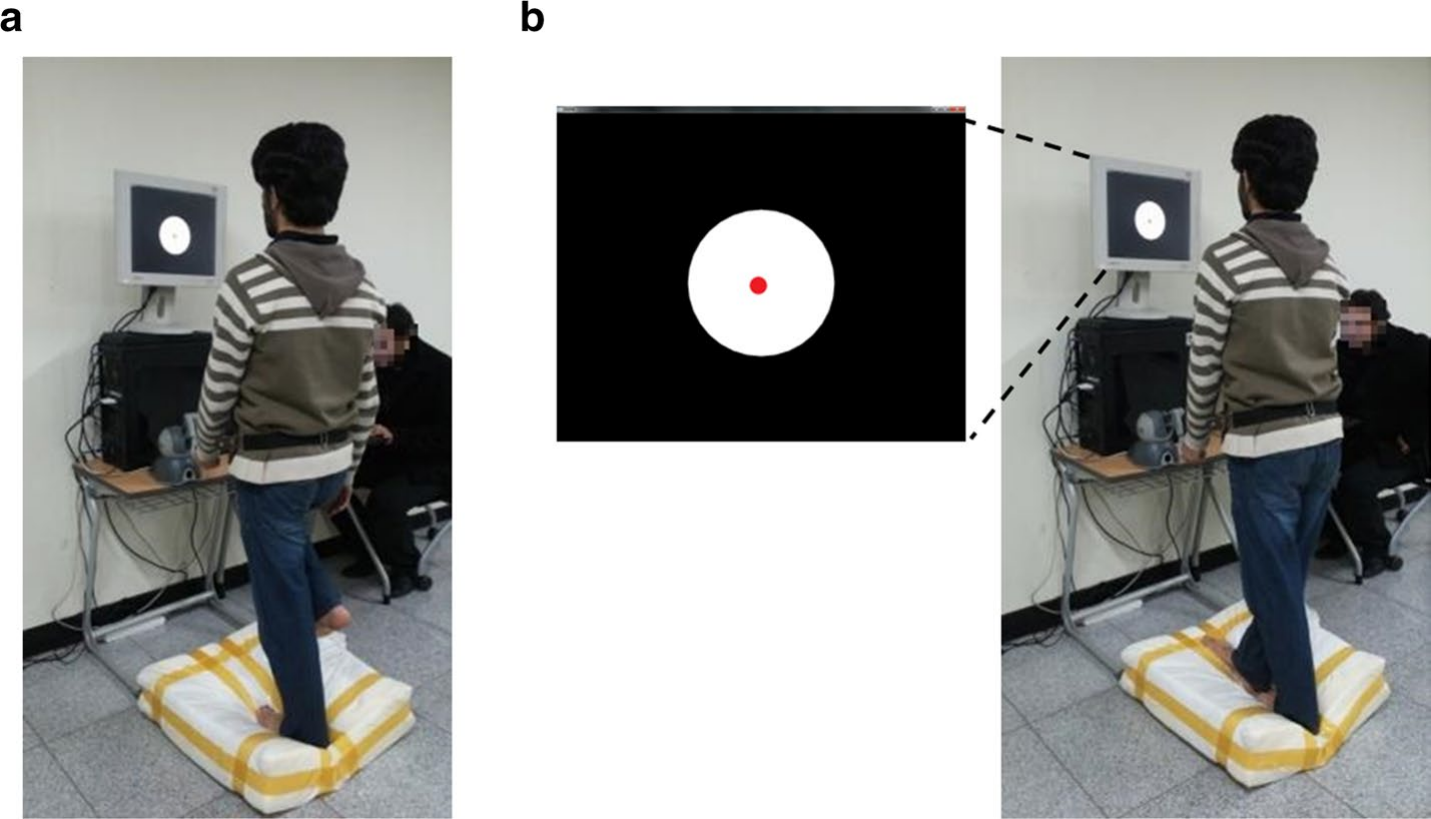
Postures assumed by young healthy subjects. **a** One foot stance P1, **b** Tandem Romberg stance P2 and (zoomed image) Visual biofeedback on display screen. Young healthy participants performed balance with assumption of each posture barefoot for 30 s. Subjects were required to stand still in front of a table, upon which the experimental apparatus including the Phantom Omni^®^ and the PC was placed

In order to experimentally test the effects of multimodal biofeedback, the subjects were asked to try and maintain their balance while standing barefoot in prescribed postures on a platform made up of foam for a period of 30 s. The platform had the dimensions of 600 × 600 × 150 mm. High resilience foam with density of 48 kg/m^3^ and tensile strength of 83 kPa was used to simulate soft ground conditions. The young healthy subjects were required to assume two distinct postures while standing on the platform, standing on one foot stance (P1) and the tandem Romberg stance (P2) as shown in Fig. 2.

Four conditions of biofeedback were applied for each subject during each posture. In the condition of no biofeedback (F1) subjects used their natural balancing capabilities to maintain upright stance, furthermore, in condition of haptic biofeedback (F2) subjects hold the phantom Omni device’s handle to get balance cues. The other three conditions consisted of different possibilities of biofeedback. In the condition of haptic biofeedback (F2) subjects held the phantom handle of the Omni device to obtain balance cues, likewise, in condition of visual biofeedback (F3) subjects utilized the visual cues provided through the display screen to balance themselves, and lastly in the condition of multimodal biofeedback (F4), the subjects utilized both haptic and visual biofeedback simultaneously to achieve their objective.

Appropriate utilization of the haptic and visual biofeedback for assistance in balance control was explained to all subjects. The surrounding environment was designed to lack any stimulus. Subjects were instructed to remain silent. 60 s of rest time was provided to all subjects between trials on each condition. Selection of posture and biofeedback was random for young healthy subjects.

All trunk tilt values of ML and AP values were analyzed using the MATLAB^®^ software. Projection of trunk tilt (PT) was calculated from the data of trunk tilt angles and smartphone’s attachment height, given by Eqs. (3) and (4):

3 $$ PT_{ML} = trunk\,til{t_{ML}}*h\quad ({\text{cm}}) $$

4 $$ PT_{AP} = trunk\,til{t_{AP}}*h\quad ({\text{cm}}) $$

where “h” is the height of smartphone’s attachment to the subject’s trunk from ground up. Since the tilt angles are small, PT can be linearized as Eqs. (3) and (4). Similar to our approach, other researchers have used trunk tilt projection derived from an electromagnetic sensor; identified balance and stability behavior, and classified individuals on the basis of age, gender, height and weight [28–31]. Mean velocity displacement (MVD), planar deviation (PD), ML Trajectory (MLT) and AP (APT) Trajectory was calculated as parameters of body sway using Eqs. (5)–(8):

5 $$ {\text{MVD}} = \frac{{\mathop \sum \nolimits \frac{{\sqrt {\left( {\left( {{\text{PT}}_{\text{ML}} ({\text{i}}) - {\text{PT}}_{\text{ML}} ({\text{i}} - 1)} \right)^{2} + \left( {{\text{PT}}_{\text{AP}} ({\text{i}}) - {\text{PT}}_{\text{AP}} ({\text{i}} - 1)} \right)^{2} } \right)} }}{{{\text{t}}_{\text{i}} - {\text{t}}_{{{\text{i}} - 1}} }}}}{\text{n}}\quad ({\text{cm}}/{\text{s}}) $$

6 $$  {\text{PD}} = \sqrt {{{\upsigma }}^{2} {\text{PT}}_{{{\text{ML}}}}  + {{\upsigma }}^{2} {\text{PT}}_{{{\text{AP}}}} } \quad ({\text{cm}})  $$

7 $$ {\text{MLT}} = \sum \left| {{\text{PT}}_{\text{ML}} \left( {{\text{i}} + 1} \right) - {\text{PT}}_{\text{ML}} \left( {\text{i}} \right)} \right|\quad ({\text{cm}}) $$

8 $$ APT = \sum \left| {{\text{PT}}_{\text{AP}} \left( {{\text{i}} + 1} \right) - {\text{PT}}_{\text{AP}} \left( {\text{i}} \right)} \right|\quad ({\text{cm}}) $$

where “i” is the index of tilt data, “n” is the total number of data values and “t” is time. MVD is the mean value of all PT velocities; changes in the ML and AP are combined to yield a single velocity value. PD is defined as the square root of sum of variances (*σ*^2^) of PT displacement in ML and AP directions. Variance of PT displacement measures show how far the PT is spread out. Similarly the sums of changes in ML and AP projection of tilt yield MLT and APT, respectively. A larger value of these parameters indicates the greater balance difficulty. A two-way analysis of variance (ANOVA) was used to identify the interaction effects of postures (one foot stance, tandem Romberg stance) and biofeedback (no feedback, haptic, visual, and multimodal) on body sway. Furthermore, main effects were analyzed using one-way analysis of variance (ANOVA) and Tukey’s HSD test was used for post hoc analysis.

## Results

The means and standard deviations (SD) of data from the experimental trials show that provision of biofeedback reduced the body sway in both the one foot and tandem Romberg postures (Table 1). Reduction in body sway can be deduced with the analysis parameters [Eqs. (5)–(8)]. The mean values of F2 (haptic biofeedback), F3 (visual biofeedback) and F4 (multimodal biofeedback) are smaller compared to F1 (no biofeedback) which indicates that on provision of biofeedback young healthy subjects gained positive contributions to their perception of balance and hence reduced the body sway to a significant and observable level. Two-way ANOVA results revealed that main effect biofeedback was statistically significant (MVD [F(3, 80) = 6.51, p < 0.001], PD [F(3, 80) = 4.79, p = 0.004], MLT [F(3, 80) = 6.76, p < 0.001], APT [F(3, 80) = 2.87, p = 0.001]) and main effect posture was not statistically significant (MVD [F(1, 80) = 0.002, p = 0.963], PD [F(1, 80) = 0.46, p = 0.498], MLT [F(1, 80) = 0.77, p = 0.380], APT [F(1, 80) = 1.54, p = 0.219]). Two-way ANOVA results also displayed no statistically significant interaction between the effects of postures and biofeedback on body sway analysis parameters (MVD [F(3, 80) = 0.78, p = 0.508], PD [F(3, 80) = 0.24, p = 0.867], MLT [F(3, 80) = 0.59, p = 0.619] and APT [F(3, 80) = 1.09, p = 0.358]).

**Table 1:** Results of body sway among young healthy subjects. Provision of biofeedback reduced the body sway

Analysis parameter	Posture	Biofeedback							
		F1		F2		F3		F4	
		Mean	SD	Mean	SD	Mean	SD	Mean	SD
MVD (cm/s)	P1	0.716	0.229	0.474	0.096	0.517	0.075	0.459	0.086
	P2	0.805	0.328	0.383	0.124	0.576	0.126	0.411	0.087
PD (cm)	P1	2.840	1.891	2.031	0.596	1.781	0.838	1.439	0.321
	P2	3.387	1.621	2.123	1.061	2.173	1.120	1.281	0.271
MLT (cm)	P1	156.1	42.49	96.68	25.57	113.7	14.17	91.44	28.07
	P2	165.0	71.55	73.54	16.72	113.2	28.46	70.09	16.76
APT (cm)	P1	115.0	45.83	83.80	16.67	81.52	18.03	83.23	10.11
	P2	142.1	53.07	72.668	33.98	107.7	23.01	85.57	18.67

Mean and standard deviation (SD) are shown here. Reduction in body sway can be deduced with the comparative analysis of parameters [Eqs. (5)–(8)]

No significant interaction between posture and biofeedback led to post hoc analysis of postures (P1 and P2) separately. Tables 2 and 3 illustrate post hoc analysis results of one foot stance (P1) and tandem-Romberg stance (P2) respectively.

**Table 2:** Results post hoc analysis results in postural condition of one foot stance

Analysis parameter	Comparison of biofeedback (p value obtained from post hoc analysis)					
	F1 vs F2	F1 vs F3	F1 vs F4	F2 vs F3	F2 vs F4	F3 vs F4
MVD	0.028	0.047	0.019	0.946	0.997	0.881
PD	0.581	0.357	0.149	0.978	0.782	0.947
MLT	0.010	0.049	0.005	0.748	0.989	0.565
APT	0.206	0.159	0.193	0.998	0.999	0.999

Tukey’s HSD test was used for post hoc analysis

F1: no biofeedback, F2: haptic biofeedback, F3: visual biofeedback, F4: multimodal biofeedback. p values are presented from the results of post hoc analysis

**Table 3:** Results post hoc analysis results in postural condition of tandem Romberg stance

Analysis parameter	Comparison of biofeedback (p value obtained from post hoc analysis)					
	F1 vs F2	F1 vs F3	F1 vs F4	F2 vs F3	F2 vs F4	F3 vs F4
MVD	0.005	0.199	0.009	0.328	0.994	0.462
PD	0.242	0.274	0.0198	0.999	0.577	0.530
MLT	0.004	0.149	0.002	0.346	0.998	0.278
APT	0.012	0.344	0.048	0.327	0.917	0.690

Tukey’s HSD test was used for post hoc analysis

F1: no biofeedback, F2: haptic biofeedback, F3: visual biofeedback, F4: multimodal biofeedback. p values are presented from the results of post hoc analysis

**Fig. 3:**
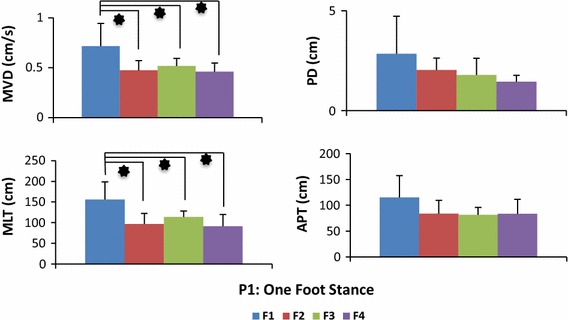
Body sway of young healthy subjects in one foot stance. Participants performed balance task of one leg stance in four biofeedback conditions: no feedback (F1), haptic biofeedback (F2), visual biofeedback (F3) and multimodal biofeedback (F4). *p value < 0.05

**Fig. 4:**
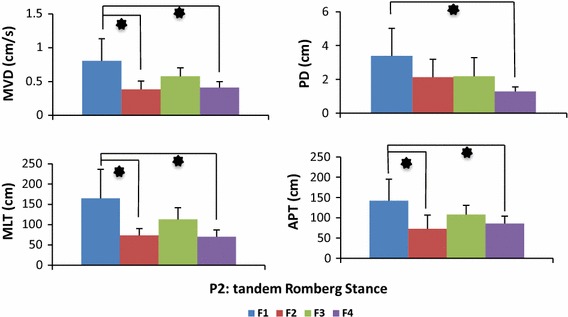
Body sway of young healthy subjects in tandem Romberg stance. Participants performed tandem Romberg stance balance task under four biofeedback conditions: no feedback (F1), haptic biofeedback (F2), visual biofeedback (F3) and multimodal biofeedback (F4). *p value < 0.05

All subjects exhibited maximum body sway during no biofeedback trials, followed by either haptic or visual biofeedback and in most of the trials the multimodal biofeedback of visual and haptic together produced the minimum amount of body sway. Multimodal (F4), visual (F3) and haptic(F2) biofeedback provided significant improvement of balance as compared to no feedback mode (F1), however no significant differences were found among comparison with each other (F2 vs F3, F2 vs F4, F3 vs F4). Figures 3 and 4 shows extent of body sway in different forms of biofeedback during one foot stance and tandem Romberg stance, respectively.

## Discussion

Body sway is a meaningful indication, which can be used to recognize the balance of a human being during upright standing posture [10]. Biofeedback based system provides the compromised sensory information to retrain sensorimotor function [32]. In this paper a novel system was presented which can provide the customized training of balance based on either visual biofeedback or haptic biofeedback or combined haptic and visual multimodal biofeedback.

Experiments conducted on young healthy subjects supported the credibility of the system. Trial results show that when subjects utilized biofeedback a reduction in body sway was achieved. The no biofeedback condition (F1) was utilized as baseline to compare it with biofeedback conditions. Haptic biofeedback (F2) effectively contributed (P1: MVD [F(3, 40) = 3.36, p = 0.028], MLT [F(3, 40) = 4.03, p = 0.010] and P2: MVD [F(3, 40) = 4.97, p = 0.005], MLT [F(3, 40) = 5.19, p = 0.004], APT [F(3, 40) = 4.14, p = 0.012]) in assisting young healthy subjects, similar results were obtained in our previous study about effects of haptic biofeedback on standing stability [24]. Visual biofeedback (F3) contributed in reduction of body sway during P1 and P2 but was statistically significant only in reducing MVD [F(3, 40) = 2.89, p = 0.047] and MLT [F(3, 40) = 2.85, p = 0.049] while assuming P1. Research study [17], demonstrated significant reduction of body sway among young healthy subjects with visual biofeedback application in a much comfortable upright standing posture than the one we used in our study. As hypothesized, significant reduction (p < 0.05) was observed amongst most of the analysis parameters on a provision of multimodal biofeedback (F4). All young healthy subjects reduced body sway significantly with the application of multimodal biofeedback (P1: MVD [F(3, 40) = 3.71, p = 0.019], MLT [F(3, 40) = 4.97, p = 0.005], and P2: MVD [F(3, 40) = 4.41, p = 0.009], PD [F(3, 40) = 3.67, p = 0.019], MLT [F(3, 40) = 5.88, p = 0.002], APT [F(3, 40) = 2.87, p = 0.048]). Theoretically, the positive effect of multimodal learning originates from a reduction of the cognitive load due to a distribution of information processing. Multiple resource theory suggests that the redundancy provided by multimodal feedback should improve performance in comparison to single-mode feedback [32, 33].

Trial results express that a multimodal biofeedback is found to be more effective in comparison with lone biofeedback of visual or haptic, this outcome commemorate the fact that our body balances with help of a multi-sensing input [10] and comprehensive augmented biofeedback could conjugate with missing capabilities [34]. However, reduction of body sway compared between multimodal (F4), visual (F3) and haptic (F2) biofeedback was not significantly different. Similarly, a multimodal (visual and vibrotactile) biofeedback system [22] also shows no substantial improvements in performance of balancing tasks when multimodal biofeedback was compared with single-mode biofeedback (visual or vibrotactile). On contrary, multimodal (visual and vibrotactile) biofeedback has proven to be effective in long-term balance training program [21]. Hence, the assessment of long-term balance training by using the novel multimodal biofeedback system presented in this research is mandatory.

Our study focused on comparing the effects of various biofeedback modalities and their comparison. We did not evaluate the long-term effects of training with the biofeedback system. The smartphone positioning on the trunk did not allow us to identify the body motions other than trunk tilt. Furthermore, no constraint was applied on the upper arm for application of biofeedback through haptic device. However, the pre-experimental instructions to the subjects overcame this limitation. Worthy conclusions can be drawn on the effectiveness of multimodal biofeedback based balance training system after further exploration and improvement.

## Conclusion and future work

Our study shows importance of multimodal biofeedback in helping the young healthy subjects to regain balance. Our balance training system points towards an important addition to the rehabilitation procedures. From this current research, analyzing the effects of multimodal biofeedback on body sway reduction of young healthy subjects would be used as a norm. In the future, we will use our system to perform a long-term balance training protocol for individuals with upright balance issues such as elderly adult, Stroke and multiple sclerosis (MS).

## Abbreviations

PC: personal computer
ML: mediolateral
AP: anteroposterior
MVD: mean velocity displacement
PD: planar deviation
MLT: mediolateral trajectory
APT: anteroposterior trajectory
ANOVA: analysis of variance
SD: standard deviation
PT: projection of trunk tilt
MS: multiple sclerosis
